# Predictors of face mask use during the COVID-19 pandemic in Indonesia: Application of the health belief model, psychological distress and health motivation

**DOI:** 10.12688/f1000research.123583.3

**Published:** 2023-12-18

**Authors:** Devi Wulandari, Fredrick Dermawan Purba, Alfikalia Alfikalia, Fatchiah Ekowati Kertamuda, Tia Rahmania, Olivia Ayu Sabrina, Kurnia Nurul Hidayah, Syarifah Fatimah

**Affiliations:** 1 Department of Psychology, Paramadina University, Mampang, Jakarta, 12790, Indonesia; 2 Faculty of Psychology, Padjadjaran University, Sumedang, West Java, 45363, Indonesia

**Keywords:** health belief model, face mask use, COVID 19, health motivation, psychological distress

## Abstract

**Background:**

High infection rates of COVID-19 in Indonesia require attention, especially transmission and prevention behaviors. One way to lower infection rates is the use of face masks. However, people’s adherence to its usage when in public is still low. This necessitates the exploration of predictors of the use of masks to increase community compliance. This study further aims to investigate the predictors of face mask use by applying the Health Belief Model, anxiety, stress, depression, and health motivation.

**Methods:**

A total of 255 respondents from Jakarta, Bandung, Tangerang, and Banten filled out an online questionnaire. Furthermore, hierarchical multiple regression was used to detect predictors associated with face mask use.

**Results:**

The results showed that the high perceived benefits and health motivation were higher in individuals who used a face mask when in public, while those who have high perceived barriers likely do not use masks. The respondent’s level of psychological distress, including depression, anxiety, and stress were not associated with face mask use.

**Conclusions:**

These findings highlight the importance of personal appraisal regarding COVID-19 and its prevention behaviors. Comfortable mask design, and emphasizing the benefits of using masks in the community improve compliance.

## Introduction

COVID-19 received worldwide attention due to its rapid spread and its severe consequences ( Li *et al.*, 2020a). The first confirmed case occurred in Wuhan City, Hubei, China, at the end of 2019, and various countries took efforts as early as possible to suppress its spread ( WHO, 2020). This disease began to spread worldwide on January 3, 2020 ( Lathifa *et al.*, 2021). In this regard, Indonesia has the highest number of COVID-19 cases in Southeast Asia with a total of 4,026,837 in August 2021 ( Satgas COVID-19, 2021).

To cope with the COVID-19 pandemic, the Indonesian government enacted various programs on national levels. They launched the health protocols campaign that encourages people to wear a mask, wash hands, social distance, stay away from crowds, and maintain mobility ( KEMENKES RI, 2021). The government also intensified the national COVID-19 vaccination program. However, various essential obstacles will potentially hamper the process of reducing cases in Indonesia.

One of the obstacles faced by the implementation of health protocols program originates from the community. People need to be continuously reminded to implement the health protocols in their daily activities, especially in terms of wearing face masks correctly and appropriately ( Buana, 2020). The World Health Organization (WHO) (2020) stated that face mask use should become a comprehensive effort to prevent the spread of COVID-19. The virus is spread due to droplets originating from a patient’s respiratory tract within one meter. In line with Shereen *et al.* (2020), face masks are necessary and have become a new habit to be implemented outside the home (when interacting with other people). However, there is still a refusal or neglect to use face masks. In Jakarta, 10,416 people were given sanctions for violating the rules for using face masks during the week the Emergency Community Activity Restriction (PPKM) policy was implemented from 3 ^rd^-9 ^th^ July, 2021 ( Paat, 2021). Likewise, an increasing number of violations also happened in Bandung and Tangerang ( Naufal, 2021).

In a study conducted in Indonesia by Firdayanti *et al.* (2020) on the prevention of COVID-19 through community service activities, the distribution of face masks was carried out door to door. This distribution was usually accompanied by demonstrations of their use by lecturers, students, community leaders, religious leaders, or local government officers. This was in line with Li *et al.* (2020b) showing that the effective use of masks combined with social distancing was successful in flattening the curve of the pandemic. Additionally, Brooks & Butler (2021) stated that using face masks in the community can substantially reduce the transmission of the acute respiratory syndrome coronavirus 2 (SARS-CoV-2) in two ways. First, face mask use prevents infected people from spreading SARS-CoV-2 by blocking respiratory droplets containing the virus from entering the air, also known as source control. This aspect is essential as it is estimated that 50%+ of COVID-19 transmission comes from people who have never experienced symptoms or individuals in the presymptomatic phase. Second, masks can protect uninfected users.

Although face mask use is essential in eradicating COVID-19, people’s compliance is still low. According to John Hopkis Center for Communication Programs, the prevalence of Indonesia people was lower than 35% (2021). Sinuraya *et al.* (2018) stated that several factors predict low compliance of health protocols, including knowledge, motivation, perception, and belief in efforts to control and prevent disease and access available resources. Lathifa *et al.* (2021) also stated that the aforementioned factors determine the level of community compliance with health protocols in the era of globalization.

To determine how individuals comply with face mask use, a cognitive model might help. The Health Belief Model (HBM) was proposed to understand disease prevention behavior, especially medication adherence, in the 1950s ( Champion & Skinner, 2002). HBM has been widely used to understand behavioral change, even more often than social cognitive theory, reasoned action theory, and transtheoretical model ( Sulat *et al.*, 2018). Some of the health behaviors that have been studied using HBM include influenza prevention behavior ( Karimi *et al.*, 2016).

The HBM has four constructs: perceived severity, perceived barriers, perceived benefit, and perceived susceptibility ( Rosenstock *et al.*, 1988). Perceived severity refers to a person’s perception of the severity of the disease, while perceived barriers refer to factors that prevent a person from adopting a healthy behavior. Furthermore, perceived benefit is the perception one has on the benefits of carrying out healthy behavior, while perceived susceptibility is one’s perception of the level of vulnerability they have ( Sulat *et al.*, 2018). A person’s tendency to carry out healthy behaviors depends on the perception that a disease has a high severity, that one has the benefits of health behavior, perceived high vulnerability to the disease and does not experience significant obstacles when carrying out these behaviors ( Champion & Skinner, 2002).

Several studies have applied the HBM in predicting healthy behaviors related to the COVID-19 disease. Erawan *et al.* (2021) explained that significant positive predictors of interest in COVID-19 vaccination were perceived susceptibility and perceived severity in Indonesia. Meanwhile, in Malaysia, the perceived barrier was a significant negative predictor in this study. HBM was also able to predict intentions to receive the COVID-19 vaccine ( Wong *et al.*, 2020), COVID-19 prevention behavior in Iran Shahnazi *et al.* (2020b), and other various healthy behaviors. Results form a scoping review stated that the HBM may also be used in interventions and models that predict healthy behavior ( Sulat *et al.*, 2018).

Although HBM has already been used to predict face mask use ( Bressington *et al.*, 2020; Shahnazi *et al.*, 2020b; Zhang *et al.*, 2019), the role of health motivation and psychological distress on face mask use was still unclear. A person’s motivation to stay healthy (health motivation) can be a predictor that influences their compliance with using a mask. Health motivation comes from the Self-Determination Theory (SDT) proposed by Ryan and Deci (2017). Intrinsic motivation will promote a person to act based on interest and enjoyment. Meanwhile, extrinsic motivation will inspire a person to perform a healthy behavior to obtain the approval of others, increasing self-esteem, appreciation from others, and pressure from outside ( Ntoumanis *et al.*, 2021).

Although health protocols such as social distancing are beneficial in reducing the spread of COVID 19, they may also cause psychological distress to the Indonesian people ( Rias *et al.*, 2020). Lack of interpersonal connectedness could hinder societal obligation and in-group harmony which are important for Indonesian people ( Hudiyana *et al.*, 2022). Wolff *et al.* (2020) explained that pandemic containment requires people to cope with reduced social contact that may create lack of freedom, boredom and negative affects among people. Moreover, Seiter and Curran (2021) stated that the level of depression during the COVID-19 pandemic was negatively related to adherence to COVID-19 prevention measures.

This present study utilized the HBM as a theoretical framework. It is hypothesized that individuals with high perceived severity, high perceived susceptibility, high perceived benefit, and the low perceived barrier will tend to use face masks. Furthermore, the levels of depression, anxiety, and stress will be associated with low use of masks. With a more thorough understanding of the related factors, the government will be able to develop various intervention programs to increase public compliance with the use of masks. This study aimed to further determine the role of the Health Belief Model (HBM) construct, stress, depression, anxiety, and health motivation on the use of masks after controlling for demographic factors in the cities of Jakarta, Bandung, Tangerang, and Banten in Indonesia.

## Methods

### Study design

This study was conducted using a cross-sectional research design from September 2020 – February 2021 in Indonesia during the outbreak of COVID-19.

### Sampling

The respondent criteria were as follows: must be at least 18 years old, must have a minimum of a junior high school education, must live in DKI Jakarta, Banten, or Bandung areas.

A non-probability sampling method was used to gather a sample of Indonesian people who lived in Jakarta, Banten, and Bandung. Non-probability sampling is a type of sampling that is used because the sample is available, convenient and meet sample criteria ( Plano Clark & Creswell, 2015).

### Data collection

Data collection was done using the SurveyMonkey online platform. We composed an announcement consisting of general information about the study, inclusion criteria, and the study link. We circulated this announcement on discussion forums, college students’ groups and community peer groups via several channels: social media accounts of the research team (e.g., Facebook, Instagram) and instant messenger applications (e.g., WhatsApp, Line, Facebook Messenger). People who were interested in participating then clicked on a link at the bottom of the announcement and were directed to the online questionnaire. The respondents filled in all questions after agreeing to the informed consent form included in the online questionnaire. A total number of 255 respondents were recruited from December 11, 2020 – February 25, 2021. A post hoc analysis was conducted using G*Power version 3.1.9.7 based on the data from the study. With a 95% confidence interval, 12 predictors the power of the study was >0.99. Therefore, the number of sample of the study was sufficient.

### Ethics statement

The Ethics Committee of Padjadjaran University approved this research (approval number: 430/UN6.KEP/EC/2020). Informed written consent was obtained from participants and they were assured that only researchers would have access to the data and it would only be used for research purposes. Participants could complete the questionnaire anonymously.

### Instruments

The constructs of the HBM were measured by instruments developed by the researchers. An open-ended questionnaire in Bahasa Indonesia was distributed to 30 respondents as an initial step in developing the questionnaire and statements that had the most responses were included in the HBM questionnaire. Then, the early version of the HBM questionnaire was tested in the pilot study. The measures were rated on a four-point Likert scale. The scoring was from 1 (strongly disagree) to 4 (strongly agree) with a total number of 24 items. There were five items for the perceived barrier such as “wearing a mask makes it difficult for me to breathe”; five items for perceived benefit including “I feel safer if wearing a mask”; five items for perceived susceptibility, such as “I have low body resistance”; five items perceived severity, such as “COVID-19 causes death”; and four health motivation items, including “health is the main thing for me”. Three items measured face mask use behavior, for instance, “I never forget to use a face mask every time I go outside”. Furthermore, a validity analysis was carried out using the content validity method with the help of two experts in the fields of health and psychology. Internal consistency for these instruments was calculated. These instruments were proven reliable ( Cronbach, 1951), shown by Cronbach Alpha values of 0.81, 0.89, 0.81, 0.64 and 0.70 for the perceived barrier scale, perceived benefit scale, perceived susceptibility scale, perceived severity scale, and mask use behavior, respectively.

The DASS-18 was used to measure depression, stress, and anxiety ( Lovibond and Lovibond, 1995). DASS-18 in Bahasa Indonesia was developed by Damanik (2006). The questionnaire was tested for its psychometric properties. Six items measured depression, such as “I feel my life is meaningless”; six items of anxiety, including “I feel my hands are shaking”; and six items measured stress, involving “I tend to overreact to certain situations”. For samples of this study, DASS-18 was shown as reliable, with Cronbach’s Alpha values of 0.81, 0.79, and 0.70 for depression, anxiety, and stress scale, respectively. A copy of the questionnaire can be found under *Extended data* ( Wulandari *et al.*, 2022).

### Data analysis

Data was analyzed using SPSS 25. Descriptive statistics were carried out for respondents’ demographic analysis. Afterwards, the relationships between sociodemographic variables, HBM variables and face mask use were evaluated using Pearson Product Moment and T-Test. Hierarchical multiple regression analysis was also carried out to examine the role of predictors based on HBM regarding mask-use behavior.

## Results

Based on the data in Table 1, the respondents mean age was 33.1 years old (SD=11.9), the majority were females (72.6%), had a university-level education (69.4%), and more than half had a monthly income of IDR 2.5 million and above and were living in urban areas (65.6%). Note that Several respondents did not complete part of their demographic data, therefore the sum of each demographic characteristics were not equal to 255.

**Table 1.** Demographic data of respondents (N=255).

Demographic data	Mean	SD
Age *	33.1	11.9
	**N**	**%**
Gender *		
Male	69	27.4
Female	183	72.6
Education *		
Junior High School	1	4.0
Senior High School	76	30.2
University	175	69.4
Income per Month (in IDR) *		
No income	77	30.3
Below 500,000	11	4.3
500,000 – 1,000,000	14	5.5
1,000,001 – 2,500,000	19	7.5
2,500,001 – 5,000,000	38	15.0
5,000,001 – 10,000,000	42	16.5
Above 10,000,000	53	20.9
Residence *		
Urban	166	65.6
Rural	87	34.4

* Several respondents did not complete part of their demographic data, therefore the sum were not equals to 255.

Table 2 shows the correlation between the psychological variables. The face mask use has a significantly positive correlation with the perceived benefits (r=0.55, P<0.001) and perceived severity (r=0.24, P<0.001). A negative correlation was observed in the association between face mask use and the perceived barrier (r=0.38, P<0.001).

**Table 2.** Correlation between research variables.

Variable	1.	2.	3.	4.	5.	6.	7.	8.
1. Perceived Benefit	-	0.09	0.38 **	-0.32 **	0.55 **	-0.06	-0.07	-0.07
2. Perceived Susceptibility	0.09	-	0.10	0.07	-0.03	0.20 **	0.15 **	0.15 **
3. Perceived Severity	0.38 **	0.10	-	-0.11	0.24 **	0.09	0.10	0.01
4. Perceived Barrier	-0.32 **	0.07	-0.11	-	-0.38 **	0.22 **	0.27 **	0.17 **
5. Face Mask Use	0.55 **	-0.03	0.24 **	-0.38 **	-	-0.05	-0.01	-0.04
6. Anxiety	-0.06	0.20 **	0.09	0.22 **	-0.05	-	0.64 **	0.71 **
7. Depression	-0.07	0.15 **	0.10	0.27 **	-0.01	0.64 **	-	0.67 **
8. Stress	-0.07	0.15 **	0.01	0.17 **	-0.04	0.71 **	0.67 **	-

* P-value<0.05.

** P-value<0.01.

Table 3 shows the results of multiple regression analysis that examined the role of predictors based on HBM on mask-use behavior. In step 1, the respondents’ sociodemographic variables significantly contributed to the variance of their behavior of using a mask (R ^2^=10%; F (4.233)=6.09, P<0.001)) with gender as significant predictor. In step 2, after including the HBM variables, this model was proven to significantly predict face mask use, (R ^2^=53%; F (9.228)=28.70, P<0.001). Therefore, the addition of variables based on HBM adds to the variance explained by the model by 43.7%. Stress, anxiety, and depression were included in step 3 and showed that all variables were able to explain the variance of mask use behavior of 54% (F (12.225)=21.55, P<0.001). Additionally, perceived barriers were a negative predictor of face mask use, while positive predictors were health motivation and perceived benefits.

**Table 3.** Summary of hierarchical regression analysis of face mask use.

Variable	*β*	*T*	*R*	*R ^2^*	∆ *R*
Step 1			0.31	0.10	0.08
Income per month	0.02	0.37			
Gender	0.20	4.02 ***			
Education	0.02	0.33			
Age	0.10	1.53			
Step 2			0.73	0.53	0.51
Health Motivation	0.48	8.59 ***			
Perceived Susceptibility	-0.03	-0.58			
Perceived Barrier	-0.16	-3.03 **			
Perceived Severity	-0.06	-1.16			
Perceived Benefit	-0.24	4.19 ***			
Step 3			0.73	0.54	0.51
Anxiety	-0.05	-0.66			
Depression	0.02	0.35			
Stress	0.07	1.15			

N=255.

^*^ P-value<0.05.

^**^ P-value<0.01.

^***^ P-value<0.001.

## Discussion

This study aimed to investigate the predictors of compliance of face mask use by applying the HBM, health motivation, anxiety, stress, and depression. The results showed that predictors that contributed significantly to the behavior of using masks are health motivation, perceived benefit, perceived barrier and gender. Therefore, these results indicate that when combined with the health belief model predictors, health motivation, which is intrinsic from within, possesses the most significant contribution. From the HBM predictors, only perceived benefits and perceived barriers contributed significantly to the use of masks during the COVID-19 pandemic. Gender was also a significant predictor of mask use, with women being more likely to wear masks than men. Furthermore, depression, anxiety, and stress were not proven to be significant predictors of face mask use after controlling for demographic and other psychological variables. Results of the study has shade some lights on the interaction between psychological variables regarding face mask use.

The Social Determination Theory (SDT) hypothesized that the psychological condition essential to make a change is having an autonomous motivation and assessing that one is competent to make changes or a behavior ( Ryan & Deci, 2017). Regarding SDT, autonomous motivation is required from the individual to make someone bring up behavior related to their health, such as using a mask to prevent the spread of the COVID-19 virus. The SDT argues that behavior based on autonomous motivation comes from within and is a self-expression of the individual. Therefore, individuals were willing and agreed voluntarily to perform the behavior. The autonomous motivation intrinsic to healthy living shows that individuals perceive that they have personal responsibility for their health, which promotes them to live a healthy life, in this case using masks, during the COVID-19 pandemic. Based on the results, the SDT was proven to have the most significant contribution to the behavior of using masks.

Intrinsic health motivation as a predictor of face mask use was in line with Hartmann *et al.* (2015), which shows that autonomous motivation, compared to introjected and external motivation, is the only predictor of healthy food consumption within a year. Intrinsic health motivation could predict weight management, choosing healthy foods, and having vigorous physical exercise in their spare time in one year.

Chan *et al.* (2014) explained that emphasizing individual personal values, initiating own face mask use and showing concern and understanding that wearing masks can be uncomfortable or cause difficulties, is positively correlated with the support of perceived autonomy. The perceived support for autonomy then significantly contributed positively to intrinsic autonomous motivation. Chan *et al.* (2014) observed that autonomous motivation initiates TPB indicators, which signifies that autonomous motivation plays a role as a driver in explaining not only behavioral intentions but also to the behavior around face mask use.

Of the four HBM predictors, namely perceived benefit, perceived susceptibility, perceived severity, and perceived barrier, only perceived benefit and perceived barrier contributed significantly to face mask use. However, HBM as a model can be used to explain face mask use. This study agrees with similar studies in Indonesia conducted by Winarti *et al.* (2021), who examined the effect of knowledge and determinants of HBM on face mask use.

The significant positive contribution of the perceived benefit shows that the positive perception that face mask use benefits respondents, helps to prevent the transmission of COVID-19 by promoting them to wear it more often. These results are in line with research conducted in Iran ( Shahnazi *et al.*, 2020a), where the perceived benefit was a significant predictor of COVID-19 prevention behavior. Similarly, respondents believe that the benefits of action will be related to their willingness to take action and preventive efforts to deal with the COVID-19 pandemic. As in Iran, research in Hong Kong ( Lee *et al.*, 2021) showed similar results that the more respondents perceive the benefits/advantages of using masks, the more willing they are to use masks. Another potential perceived benefit could be avoiding government sanctions. The results of a survey on 90, 967 respondents on the behavior of the Indonesian people during the COVID-19 pandemic (7-14 September 2020) (Central Statistics Agency, 2020) showed that more than half of the respondents stated that the reason for not implementing health protocols was because there were no sanctions. Related to the results of the BPS research (2020) and this research, it could be that apart from the belief that wearing a mask can reduce the threat of contracting the COVID-19 disease, one of the other benefits that are strongly felt from face mask use during the COVID-19 pandemic was not being subject to the threat of sanctions when wearing a mask in certain locations.

The results of this study indicate that the perceived barrier provides a significant negative contribution to using masks. Therefore, it shows that factors considered obstacles are believed to make respondents more unwilling to use their masks. The results of a survey of the Indonesian people from the BPS (2020) showed that there were several reasons why respondents did not apply health protocols ranging from the highest to the lowest results, namely: the absence of sanctions if they did not apply the health protocols; the absence of COVID-19 cases in the surrounding environment; work becomes difficult if you have to apply health protocols due to difficulties in social interactions and the price of masks, face-shields, hand sanitizers or PPE which tends to be expensive; other people were not using face masks and officials or leaders not setting an example. A person’s tendency to carry out healthy behaviors will be higher if they perceive less significant obstacles when enacting healthy behaviors ( Champion & Skinner, 2002). In this case, several factors observed from the BPS survey (2020) made the barrier to not wearing masks appear to be more related to social context, rather than health related factors.

Another result of this study is that the perceived severity does not have a significant effect but still positively correlates with using masks. Therefore, the more respondents perceive the high severity of COVID-19, the more favorable it will be. In line with Lee *et al.* (2021), the higher the confidence of research respondents on the seriousness of COVID-19 disease, the more willing they are to display the behavior of wearing masks.

Perceived susceptibility does not have a significant contribution to the behavior of using masks. This variable is not even significantly correlated with the behavior of using masks. As previously stated, perceived susceptibility refers to a person’s assessment of their level of vulnerability to a particular disease ( Sulat *et al.*, 2018). Champion & Skinner (2002) also explain that a person’s tendency to carry out healthy behavior depends on their perception of a disease’s severity and vulnerability.

Furthermore, an analysis of the survey responses regarding whether family members, neighbors, and co-workers/friends on campus were positive for COVID-19, shows that not many people are affected by COVID-19 in the environment close to the respondent. Therefore, it is plausible that most respondents do not consider themselves and the people immediate around them vulnerable to the disease. These results may explain the absence of a significant contribution of perceived susceptibility to mask-wearing behavior.

This study showed that depression, anxiety, and stress were not significant determinants for face mask use. There were also no significant correlations between depression, anxiety, and stress to face mask use. This result was in line with Wang *et al.* (2020) who studied the association between mental health and face mask use during the COVID-19 pandemic in Poland and China. Wang *et al.* (2020) showed that depression, anxiety, and stress were not significant determinants of face mask use in both Poland and China. Depression, anxiety and stress in the study were measured as general concept, not focusing on contamination of COVID 19, therefore, it is plausible these variables is not directly correlated with face mask use. The results of this present study were in contrast with Xu *et al.* (2022) that showed the association between anxiety symptoms and face mask use in junior and senior high school students in China. Bressington *et al.*’s (2020) study in Hong Kong also showed that respondents with higher frequency of reusing masks, wearing face masks for self-protection, and the perceived high severity of COVID-19 were more likely to report depressive symptoms. There were 46.5% of respondents in the Bressington *et al.* study that were reported to have probable depression, which was high compared to the previous study in Hong Kong.

Compared to the study by Bressington *et al.* (2020), respondents in this research can be categorized as low in depression, anxiety, and stress as the mean scores were 3.33, 2.76, and 2.55 respectively. These scores can be considered low when compared to the maximum score of each DASS Scale which was 21. The low level of depression, anxiety, and stress experienced by our respondents may explains why there was no significant contributions of depression, anxiety, and stress to face mask use.

There were several limitations in this study: first, the sampling strategy being used was non-random, thus the representativeness of the population and generalizability of the study may be compromised. The strategy of using instant messaging and social media apps to reach potential respondents was potentially biased to include mostly middle-to-high socioeconomic statuses and the level of education of respondents. Therefore, any generalization to other populations should be conducted cautiously. Second, cross-sectional design of the study might not allowed for explaining cause and effect between variables. Thus, a prospective study design may be applied in the further study to ascertain cause and effect relationship between variables.

## Conclusions

In Indonesia, wearing a face mask is a highly important strategy in reducing the spread of COVID-19. This study showed that health motivation and the perceived benefit increased mask use, while perceived barrier decreased it. Therefore, these results shed light on the importance of human motivation regarding the infection and prevention of COVID-19. Although several countries allow not to using face mask in public area due to the less harmful COVID 19 variant, face mask use is still important for future prevention of any other dangerous airborne disease from spreading. Thus, the behavioral intervention to increase people’s compliance in face mask use should emphasize the benefits to their health and work to design more comfortable face masks.

## Data availability

### Underlying data

The underlying data for this research is available on request for research purposes only. To access the data, please contact the corresponding author Devi Wulandari ( devi.wulandari@paramadina.ac.id). Any researcher interested in the data must send their research proposal and proof of affiliation.

### Extended data

Figshare: Questionnaire for Face Mask Use Behavior, DASS and Health Belief Model. https://doi.org/10.6084/m9.figshare.20763226.v1 ( Wulandari *et al.*, 2022).

This project contains the following extended data:

- Questionnaire

Data are available under the terms of the Creative Commons Zero “No rights reserved” data waiver (CC0 1.0 Public domain dedication)
