# Supplementary material for: Predictors of face mask use during the COVID-19 pandemic in Indonesia: Application of the health belief model, psychological distress and health motivation
Source: F1000Res. 2023 Dec 18;11:1080. Originally published 2022 Sep 21. [Version 3] doi: 10.12688/f1000research.123583.3 (PMC10687382; doi:10.12688/f1000research.123583.3)
Supplement: Peer review history

# Reviewer report: Reviewer response for version 3

**DOI:** 10.5256/f1000research.160187.r231027

**Published:** 2024-01-04

**Authors:** Jan Passchier

**Affiliations:** 1 Faculty of Behavioural and Movement Sciences, Clinical Psychology, Vrije Universiteit Amsterdam, Amsterdam, The Netherlands

The authors have adequately responded to my comments and adapted the manuscript accordingly.

 Three minor comments:

 1. Discussion: 1st paragraph: 'has shade some lights' should be 'has shed some light'

 2. 4th paragraph: I was not able to see the abbreviation 'TBP' in full words in the present manuscript. If indeed not present, please add here

 3. 12th paragraph: 'explains' should be 'explain'

Is the work clearly and accurately presented and does it cite the current literature?

Yes

If applicable, is the statistical analysis and its interpretation appropriate?

Partly

Are all the source data underlying the results available to ensure full reproducibility?

Partly

Is the study design appropriate and is the work technically sound?

Yes

Are the conclusions drawn adequately supported by the results?

Partly

Are sufficient details of methods and analysis provided to allow replication by others?

Partly

Reviewer Expertise:

Medical Psychology

I confirm that I have read this submission and believe that I have an appropriate level of expertise to confirm that it is of an acceptable scientific standard.

# Reviewer report: Reviewer response for version 3

**DOI:** 10.5256/f1000research.160187.r231026

**Published:** 2023-12-21

**Authors:** Mary Abboah-Offei

**Affiliations:** 1 Edinburgh Napier University, Edinburgh, Scotland, UK

No further comment

Is the work clearly and accurately presented and does it cite the current literature?

Yes

If applicable, is the statistical analysis and its interpretation appropriate?

I cannot comment. A qualified statistician is required.

Are all the source data underlying the results available to ensure full reproducibility?

Yes

Is the study design appropriate and is the work technically sound?

Yes

Are the conclusions drawn adequately supported by the results?

Yes

Are sufficient details of methods and analysis provided to allow replication by others?

Yes

Reviewer Expertise:

Global health

I confirm that I have read this submission and believe that I have an appropriate level of expertise to confirm that it is of an acceptable scientific standard.

# Reviewer report: Reviewer response for version 2

**DOI:** 10.5256/f1000research.143841.r221713

**Published:** 2023-11-29

**Authors:** Mary Abboah-Offei

**Affiliations:** 1 Edinburgh Napier University, Edinburgh, Scotland, UK

This is a well written manuscript that is relevant to its context and globally as fight against COVID-19 continuous.

 There are however, minor amendments required before this manuscript can be accepted for indexing:

 1. In the conclusion of the abstract, could the authors start the sentence with 'These' rather than 'Therefore'.

 2. Could the authors decide if they want to define 'Health Belief Model' (HBM) once and use the HBM consistently throughout the manuscript or just stick to Health Belief Model.

 3. In the methods section under 'sampling', could the authors check the last word 'kriteria' and amend it as appropriate or clarify the meaning.

 4. Could the authors provide some reference to support the last sentence on page 5 under 'Instruments' "These instruments were proven reliable, shown by Cronbach Alpha values of 0.81, 0.89, 0.81, 0.64 and 0.70 for the perceived barrier scale, perceived benefit scale, perceived susceptibility scale, perceived severity scale, and mask use behavior, respectively".

 Thanks.

Is the work clearly and accurately presented and does it cite the current literature?

Yes

If applicable, is the statistical analysis and its interpretation appropriate?

I cannot comment. A qualified statistician is required.

Are all the source data underlying the results available to ensure full reproducibility?

Yes

Is the study design appropriate and is the work technically sound?

Yes

Are the conclusions drawn adequately supported by the results?

Yes

Are sufficient details of methods and analysis provided to allow replication by others?

Yes

Reviewer Expertise:

Global health

I confirm that I have read this submission and believe that I have an appropriate level of expertise to confirm that it is of an acceptable scientific standard.

# Reviewer report: Reviewer response for version 2

**DOI:** 10.5256/f1000research.143841.r166421

**Published:** 2023-03-17

**Authors:** Jan Passchier

**Affiliations:** 1 Faculty of Behavioural and Movement Sciences, Clinical Psychology, Vrije Universiteit Amsterdam, Amsterdam, The Netherlands

The authors adapted their manuscript satisfactorily and in line with my previous review. A few small suggestions still remain:

- Introduction, 5th paragraph: change 'the prevalence of Indonesian people ..' in: 'it was in Indonesia'
- Methods, Sampling, last sentence: change 'kriteria' in 'criteria'.
- Discussion, 4th paragraph: mention 'TPB' also in full (Theory of Planned Behaviour).

Is the work clearly and accurately presented and does it cite the current literature?

Yes

If applicable, is the statistical analysis and its interpretation appropriate?

Partly

Are all the source data underlying the results available to ensure full reproducibility?

Partly

Is the study design appropriate and is the work technically sound?

Yes

Are the conclusions drawn adequately supported by the results?

Partly

Are sufficient details of methods and analysis provided to allow replication by others?

Partly

Reviewer Expertise:

Medical Psychology

I confirm that I have read this submission and believe that I have an appropriate level of expertise to confirm that it is of an acceptable scientific standard.

# Reviewer report: Reviewer response for version 1

**DOI:** 10.5256/f1000research.135703.r151141

**Published:** 2022-10-06

**Authors:** Jan Passchier

**Affiliations:** 1 Faculty of Behavioural and Movement Sciences, Clinical Psychology, Vrije Universiteit Amsterdam, Amsterdam, The Netherlands

General Remarks

 The manuscript (entitiled *Predictors of face mask use during the COVID-19 pandemic in Indonesia: Application of the health belief model, psychological distress and health motivation) *describes an investigation of psychological predictors of face mask use in Indonesian cities. Perceived benefits and health motivation were found to be relevant predictors. 

 The study is interesting and gives insight into the determinants of preventative behavior of COVID-19 during its most dangerous phase in a Low-Middle Income Country. The research is straightforward, with a simple cross-sectional design using a convenient sample. It is clearly written, although the English can be improved. Some discussion parts should be elaborated more than is written here. Details of my review are presented below: 

Introduction

- 1 ^st ^sentence add: 'and its severe consequences'
- 3 ^rd^ paragraph: instead of 'is a comprehensive effort', 'should become a comprehensive effort'  might be better, I think. In the sentence 'In line with ..' the word 'absolute' is not clear. 
- 4 ^th^ paragraph: Mention the country of the study of Firdayanti (probably Indonesia) Next sentence: 'that showed': better: 'showing that'. 
- 5 ^th ^paragraph: please give the figure on compliance in Indonesia if available. 
- 7 ^th^ paragraph: 'vulnerability' seems more a characteristic of a person than of a disease. 
- 8 ^th  ^paragraph: mention the countries in which the referred studies were carried out. 
- 9 ^th  ^paragraph: mention the outcome of the HBM studies. 
- 10 ^th^ paragraph: it might be mentioned that psychological distress in Indonesian people is in particular the case since social contact is very important in Eastern cultures like Indonesia. 

Methods

*Study Design*

- see general remarks

*Sampling*

- please give a short description of 'non-probability sampling method' for readers with a reference. 

*Data collection*

- perform a power analysis to demonstrate that n=255 is a sufficient sample fo analyzing the number of predictors.

*Instruments*

- The HBM version and its development should be described more. It was probably an Indonesian version? Were the items developed by the researchers or translated from existing English versions? Was the development carried out on the current-data set or another one?
- Add " " to the item COVID-19 causes death.
- The use of three decimals for the Cronbach alpha's suggests preciseness that is not realistic. Two decimals suffice. If data are available about the validity of this version please report it as well. 
- 2 ^nd^ paragraph: start with 'The' DASS etc. See further my remarks above on the HBM. 

Data Analysis

- Overall the statistical analysis used, seem appropriate. Its application on other than psychological variables (i.e. the demographic variables) should be mentioned here as well. 
- The use of 3 decimals for the Cronbach alpha's suggests an exactitude that is not realistic. Two decimals suffice.

Results 

- 1 ^st  ^paragraph: the sentence starting with 'Note' about non-response regarding the demographic data is already put under Table 1 and can be deleted in the text. The use of 3 decimals suggests an exactitude that is not realistic. Two decimals are sufficient. 
- 2 ^nd ^ paragraph: In the heading of Table 2 and the text the word 'psychological' should be added to 'variables'. 'The behavior of face mask use..': omit 'behavior of'. It would be interesting when Cohen's d's are presented for the significant correlations (and in Table 3 the regression coefficients) to see the qualification in terms of a small, moderate or large effect.
- 3 ^rd ^paragraph: Mention 'gender' as a significant predictor. 

Discussion

- 1 ^st ^Paragraph: the sentences about the predictive value of perceived benefits and barriers can be merged. I would also recommend one or more sentences stating in which respect the outcome of this study is new, respectively, add to what we already know. It is good to mention that depression, anxiety, and stress were no significant predictors when corrected for the demographic and other psychological variables. 
- 4 ^th^ paragraph: I prefer not to describe the study of Chan in detail, or otherwise to do so in the introductory section. Better to restrict yourself to which of your findings are in line with that and other studies; and which are different with an explanation for the difference. R6: add 'perceived' to 'support'. 
- 5 ^th ^paragraph: be more clear about the independent and respectively dependent contribution of the predictors. Mention which population was studied by Winarti *et al. *and if their HBM predictors were analyzed with correction for other variables or not. 
- 10 ^th ^paragraph: Last sentence: 'The analysis results ...': in the present study or in that of Champion and Skinner? 
- 11 ^​​​​​​th^ paragraph: 2 ^nd ^sentence: 'it can be concluded' is my opinion too strong 'it is plausible' might be better. 
- 12 ^th^ paragraph: It is important to note that stress, anxiety, and depression are measured as general concepts and not focused on contamination by Covid-19. 3 ^rd ^sentence: 'Some of the results' can be deleted. 'Furthermore': better changed to 'However'.
- 13 ^th^ paragraph: 'explains' is too strong: 'may explain' leaves room for other explanations.
- 15 ^th^ paragraph: an important limitation that is not mentioned here is the cross-sectional design of this study which does not allow firm conclusions about cause-effect relations between the variables. Further: explanations how the bias due to the sample selection in the present study might have influenced the outcome can be added (in particular the demographic variables, such as gender). A paragraph should be added on future research and possible application of the current results. It should also be noted that currently many measures, also face mask use are now (partly) abolished due to the dominance of a less dangerous COVID-19 variant. (Omicron).

Abstract

- I could not find the outcome on 'comfortable mask use' in the results section. 

Is the work clearly and accurately presented and does it cite the current literature?

Yes

If applicable, is the statistical analysis and its interpretation appropriate?

Partly

Are all the source data underlying the results available to ensure full reproducibility?

Partly

Is the study design appropriate and is the work technically sound?

Yes

Are the conclusions drawn adequately supported by the results?

Partly

Are sufficient details of methods and analysis provided to allow replication by others?

Partly

Reviewer Expertise:

Medical Psychology

I confirm that I have read this submission and believe that I have an appropriate level of expertise to confirm that it is of an acceptable scientific standard, however I have significant reservations, as outlined above.

# Response

**Published:** 2023-01-31

**Authors:** Devi Wulandari

**Affiliations:** Paramadina University, Indonesia

We already add several changes in the manuscript accordingly:

 Introduction:

1. We made several revisions to the mentioned sentences in paragraphs 1 ^st^, 3 ^rd^, and 7 ^th^
2. the country of the study in the 4 ^th^ paragraph was mentioned
3. We provide more data on compliance in Indonesia in paragraph 5 ^th^
4. We added the name of the country in paragraph 8 ^th^
5. We explained more about the outcome of HBM studies in paragraph 9 ^th^
6. More rationale in the 10 ^th^ paragraph was already added

Methods

1. We already gave a short description of non probability sampling method
2. We already perform power analysis to show sufficient of the sample
3. Explanation regarding HBM version were already added
4. We already added “ to the item COVID 19 causes of death
5. Tables using two decimal were already updated
6. Further explanation regarding DASS was already added in the manuscript

Data Analysis

1. We already mentioned demographic variables in the results section

Results

1. 2 ^nd^ paragraph added the word psychological. Gender as a significant predictor is already mentioned

Discussion

1. We already merged some sentences and added significance to the study and reduced the explanation from the study of Chan
2. In 5 ^th^ paragraph, the discussion regarding independent contribution was revised, and the population from the study of  Winarti et al. was already mentioned
3. 10 ^th^ paragraph: the sentence was revised
4. 11 ^th^ paragraph: We changed concluded into plausible
5. 12 ^th^ paragraph: definition of stress, anxiety and depression as a general concept was noted, and suggested revisions were already applied
6. 13 ^th^ paragraph: explains changed into may explain
7. 15 ^th^ paragraph: limitation of the study was mentioned in the article

Abstract:

1. The suggestion for comfortable face mask use was for perceived barrier results
